# Wellbeing, nature connection and vaccine attitudes: A convergent mixed methods study in Wim Hof Method practitioners

**DOI:** 10.1371/journal.pmen.0000281

**Published:** 2025-03-26

**Authors:** Jade L. Huish, Zoe Fisher, Amy Isham, Andrew H. Kemp

**Affiliations:** 1 School of Psychology, Faculty of Medicine, Health & Life Science, Swansea University, Swansea, United Kingdom; 2 Regional Neuropsychology and Community Brain Injury Service, Morriston Hospital, Swansea, United Kingdom; 3 Health and Wellbeing Academy, Faculty of Medicine, Health & Life Science, Swansea University, Swansea, United Kingdom; 4 Strategy Department, Swansea University Health Board, Baglan Head Quarters, Neath Port Talbot, United Kingdom; UNINT: Universita degli studi Internazionali di Roma, ITALY

## Abstract

Amidst global challenges like the COVID-19 pandemic and the climate crisis, there’s a pressing need for strategies that improve wellbeing. This study investigates the Wim Hof Method (WHM) as a potential tool for enhancing wellbeing and its influence on related aspects including nature connectedness and health attitudes, including vaccine uptake. We conducted a mixed-methods study involving an online survey with 192 UK-based WHM practitioners and in-depth interviews with 15 of these participants. The focus was on their wellbeing, perceptions of climate change, and decisions regarding vaccine use during the COVID pandemic. Following exclusions, a total of 132 participants were available for quantitative analysis. Findings revealed higher levels of wellbeing among WHM practitioners relative to pre-pandemic (*d*=  0.78) and pandemic-era (*d*=  1.32) benchmarks. Notably, nature connectedness was found to mediate the relationship between WHM practice and enhanced wellbeing (*p*<.05, bootstrapped). Thematic analysis yielded seven main themes: the cultivation of positive psychological states, experience of challenging climate-related emotions, improved distress management, heightened sense of connectedness, perceived vulnerability to COVID-19, moral and social responsibility, and the recognition of opportunities for positive change. Vaccine attitudes were nuanced, with some practitioners prioritising public health through vaccination while others leaned towards natural health approaches, reflecting a broader tension between individual beliefs and collective wellbeing. While 73% (96 of 132) of our sample either had received or intended to receive the vaccine, this was lower than the wider UK population at that time (96%). Overall, our findings underscore WHM’s role in not only bolstering human wellbeing during adversity but also highlight opportunities for promoting environmentally sustainable behaviours by reconnecting people to nature. This dual benefit highlights potential for fostering human flourishing as well as environmental stewardship while reinforcing the need for carefully tailored public health strategies that engage with diverse perspectives to maximise both individual and societal resilience.

Study registration

The study was registered prospectively on August 4, 2021 and registration is available here: https://doi.org/10.17605/OSF.IO/GSAE9

*“The nature outside of ourselves directly influences the nature within us. We have become strangers to nature…”* – Wim Hof, 2011, p. 323.

Major societal stressors like the COVID-19 pandemic and the unfolding climate crisis–reflected in the worsening planetary vital signs such as levels of greenhouse gasses, ocean acidity and heat, and extreme weather [1]– reveal an urgent need for strategies that enhance wellbeing and resilience across all levels of society. The pandemic, which may have arisen partly from climate-induced ecological disruptions [2] underscores a critical link between climate change and our health and wellbeing. The Wim Hof Method (WHM), a unique practice combining cold exposure, breathwork, meditation and exercise [3,4], may present an opportunity to promote human flourishing alongside collective and planetary wellbeing. Human flourishing typically refers to individual wellbeing, a multidimensional construct that integrates a focus on positive emotions (hedonia) and meaning (eudaimonia) [5]. However, wellbeing is an increasingly complex construct that also includes a focus on other people and nature [6–9]. Individual wellbeing has been argued to involve feeling good and functioning well [10], while collective wellbeing encompasses feeling good and functioning well, interdependently [11]. Planetary wellbeing aspires to “the highest attainable standard of wellbeing”, encompassing “human and non-human beings and their social and natural systems” [12]. Lomas [13] writes: “existentially, [human] wellbeing depends upon the wellbeing of the environment: Earth must be capable of supporting life for flourishing to even be conceivable…” While individuals have much greater capacity to promote individual wellbeing, they still have capacity to promote collective and planetary wellbeing, which subsequently positively impacts on individual wellbeing [6,7]. We have proposed previously that wellbeing could be defined as connection to the self, others and nature [9]. Critically, such connection may represent a fundamental psychological need, such that if that connection is not present – even if one does not desire connection – then wellbeing will be adversely impacted. WHM may provide one opportunity for realising connections to self, others and nature.

WHM has cultivated a large following, and proponents claim it can promote happiness and vitality, and even bolster a sense of connection [14]. While support for these claims is primarily anecdotal, preliminary indications suggest that such benefits may be possible. The components of WHM, including cold exposure [15], breathwork [16], meditation [17], and exercise [18] have been independently associated with higher levels of wellbeing. Often practised in natural environments, WHM may strengthen psychological connectedness to nature [19] and bolster individual wellbeing [20]. Humans have a basic psychological need for connection to the natural world, known as ‘biophilia’ [21], and studies show that people more connected to nature also feel more connected to humanity and engage in more prosocial and pro-environmental behaviours [22,23] The purported effects of WHM on wellbeing may be partly due to heightened nature connection. In other words, it is possible that nature connectedness mediates the experience of wellbeing, yet this has not been explored in past research. Published studies have focused on the physiological impacts of WHM, overlooking its effects on nature-related outcomes and psychological wellbeing [24]. To address this gap, we will explore WHM’s impact on holistic wellbeing, following a transdisciplinary model emphasising connection to the self, others, and nature [6–8], using a mixed-method study design and mediation analysis to examine whether nature connectedness mediates psychological wellbeing. This approach aims to clarify the potential benefits of WHM and its role in fostering a deeper connection to the natural world.

With its visibility in the media and high-profile celebrity endorsements, WHM has cultivated a strong online community presence and, if effective, offers much potential for collective wellbeing. Interestingly, some of the benefits discussed in online forums during the COVID-19 pandemic encompassed ‘natural’ approaches to health, raising important questions during a time when the discourse around vaccination became more polarised [25]. Research has shown that the preference for ‘natural’ over synthetic approaches to health is more pronounced among individuals who spend time in nature and feel more connected to it [26]. However, such populations are also more likely to engage in prosocial behaviour and feel a deeper connection to humanity [23], potentially supporting collective wellbeing. In engaging with the community via online platforms, a narrative was observed whereby a ‘natural’ approach to health was considered by some as inconsistent with ‘unnatural’ public health interventions such as vaccine use. This raises the question of how populations, like the WHM community, that may have a preference for natural approaches to health while also exhibiting stronger prosocial tendencies, navigate their decisions regarding vaccine use—a critical but ‘unnatural’ public health intervention. Thus, while our primary focus is to determine whether WHM enhances wellbeing and connection to nature, it is also important to explore how such practices might influence attitudes towards conventional health interventions, like the COVID-19 vaccine, and whether they complement or conflict with broader public health priorities.

Accordingly, a convergent mixed methods design is used to explore a rich and holistic understanding of wellbeing in the WHM population by reflecting on the combined insights drawn from both quantitative and qualitative data [27]. We predicted that WHM practitioners would display higher levels of individual wellbeing relative to previously published UK-based samples [28,29]. Given the associations between nature exposure, nature connectedness and wellbeing, we also sought to determine whether nature connectedness would serve as a mediating factor, potentially explaining the mechanism by which WHM influences individual wellbeing outcomes. Finally, we also investigated how wellbeing of the WHM practitioner and nature connectedness shaped their attitudes towards climate change and vaccine choices in semi-structured interviews, mindful of potential tensions between beliefs in the superiority of natural immunity and broader considerations relating to prosociality, collective health and wellbeing.

## Method

### Participants

A total of 192 UK-based WHM practitioners were recruited, of whom 132 provided sufficient data for analysis (Fig 2). This sample included 64 females (48.5%) and 68 males (51.5%), aged 20 to 75 years (M =   46.39, SD =   10.64). From this sample, a total of 15 WHM practitioners were also interviewed, including six females and nine males, aged 35 to 58 years (M =   47.53, SD = * * 6.13).

**Fig 1:**
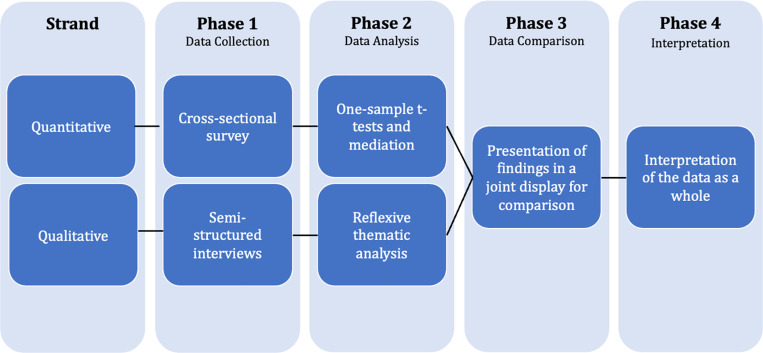
Convergent Mixed Methods Study Design.

### Ethics statement

Ethical approval was obtained from the School of Psychology ethics committee at Swansea University on 4^th^ May 2021 (Project Reference Number: 5159), and support for recruitment through WHM Facebook forums was provided by the WHM Support Team and group moderators. Formal consent was then obtained from all participants.

### Recruitment and eligibility criteria

Participants were recruited through WHM Facebook forums, following consultation with the WHM Support Team, who confirmed that these forums were an effective way to reach UK-based practitioners. In particular, the WHM UK Facebook group was identified as a key recruitment avenue due to its large and active membership and our focus on UK-based practitioners. Given the constraints imposed by the COVID-19 pandemic, online recruitment via social media was the most feasible and ethical method for engaging with this population while ensuring accessibility and minimising risk. No incentive or reward was offered for participation. WHM practitioners were invited to complete an online survey if they met the following eligibility criteria: (1) currently practise the WHM, (2) aged 18 or older, (3) live in the UK, and (4) speak English. All quantitative data was collected between the 11^th^ of May 2021 and the 5^th^ of July 2021, and selected individuals were invited for a follow-up interview, which was conducted between the 1^st^ of June 2021 and the 8^th^ of December 2021. While we had originally planned to stop conducting interviews by the 1^st^ of July for practical reasons associated with time constraints relating to an MSc project deadline, we collected additional qualitative data once the MSc project was completed to gather additional insights in a larger sample. Participants were selected for interview using maximal variation sampling, a procedure adopted to capture a wide range of practitioners’ experiences and obtain a comprehensive understanding of wellbeing in the WHM population [27,30].

### Design

A convergent mixed methods design [27] was utilised to capitalise on the strengths of both quantitative and qualitative research methods. As shown in Fig 1, this involved collecting quantitative and qualitative data concurrently, analysing them independently and examining both strands of data as a whole for convergence and divergence [27]. The quantitative component of this study adopted a cross-sectional survey design, while the qualitative phase involved collecting data using semi-structured interviews conducted via Zoom. The study protocol is available on the Open Science Framework [31].

### Materials

#### Demographics.

 Demographic data included age, sex, mental and physical health, and vaccine status.

#### WHM Practice.

Participants responded to a series of items relating to how (what pillars), when (before or during the pandemic), where (indoors, outdoors or both), and for how long (in weeks) participants had been practising.

#### Wellbeing.

Wellbeing was measured using the Warwick-Edinburgh Mental Wellbeing Scale (WEMWBS) [32] The scale includes 14 positively worded statements (e.g., “I have been feeling good about myself”) that are scored on a 5-point Likert scale. Scores range from 1 (none of the time) to 5 (all of the time). Total scores range from 14 to 70. Higher scores indicate better psychological wellbeing. The WEMWBS has good to excellent reliability (Cronbach’s α=  =  0.89 in a student sample; Cronbach’s α=  =  0.91 in a general population sample) [32]

#### Nature Connectedness.

Nature connectedness was measured using the connectedness to nature scale (CNS) [33] The scale includes 14 statements (e.g., “I often feel a kinship with animals and plants”) that are scored on a 5-point Likert scale ranging from 1 (strongly disagree) to 5 (strongly agree). Scores from each statement are totalled. However, three scale items are reverse scored (i.e., statements 4, 12 and 14). Overall scores range from 14 to 70. Higher scores indicate feeling strong emotional connections with nature. The CNS has good reliability (α= .84) [33].

#### Interview topic guide.

A topic guide was created for the interviews, and the transcripts from these interviews were used for reflexive thematic analysis. This topic guide is available in the supplementary materials (see S1 Text).

### Procedure

The online survey was made available through the Qualtrics platform, a link to which was posted on WHM Facebook forums inviting eligible participants to take part in the study. All participants were informed about the survey content and gave consent (via a tick box) before initiating the questionnaire. Demographic information and details relating to WHM practice were collected first, followed by administration of the WEMBWS and CNS. Participants were also asked to enter their email address if they were willing to participate in a follow-up interview. Using maximal variation sampling, fifteen consenting participants were selected from the survey to participate in a semi-structured interview via Zoom. The sampling procedure involved selecting participants who differed according to (1) how long they had been practising WHM, (2) where they practise WHM (i.e., indoors or outdoors), (3) their physical health status, (4) their mental health status, and (5) their vaccine status. Prior to being interviewed, each selected participant was provided with details about the interview content and was asked to provide consent in order to proceed. Only after obtaining their consent did the interview commence. All interviews, including consent, were audio-recorded using the recording feature on Zoom. All interviews were conducted by the first author of the study, and they lasted, on average, 33 minutes and ranged from 19 minutes to one hour and 24 minutes in length.

### Analysis

#### Quantitative.

Survey data were exported to SPSS (Version 28) for analysis. Incomplete, invalid and non-differentiated responses (i.e., ‘survey straightlining’) were removed prior to analysis. Outliers were also identified and removed to prevent obtaining misleading results [34]. After establishing normality, two one-sample t-tests were performed using SPSS, adopting the alpha level of.05. This study compared the wellbeing data with data from two previously published UK-based studies conducted before (N=  =  4,305) [28] and during (N=  12,989) [29] the COVID-19 pandemic. Next, after ensuring that the assumptions for mediation analysis were met [35], a simple mediation analysis (model 4) was performed using PROCESS (Version 3.5) for SPSS, adopting the alpha level of.05. The independent variable was the time spent practising WHM, with nature connectedness serving as the mediator, and the dependent variable being wellbeing. Bootstrapping, a resampling method that does not assume normality, was used to test the indirect effect, generating an empirical estimate of the sampling distribution based on 10,000 samples while enhancing the reliability and accuracy of results by reducing sample-specific biases [35]. In such analyses, if the 95% bootstrapped confidence intervals (CI) do not include 0, then it can be concluded that mediation has occurred [35]. (Refer to S2 Text for further details).

#### Qualitative.

Semi-structured interviews were audio recorded, transcribed verbatim and imported to NVivo 12 for data management. Qualitative data were analysed using reflexive thematic analysis following the guidelines suggested by Braun and Clarke [36–38]. Thematic analysis was used because it is flexible in its application regarding underlying philosophical frameworks, aligning well with the pragmatic paradigm guiding this study. Since WHM and its impact on wellbeing are relatively unexplored areas, data were coded inductively, after which findings were interpreted within the context of the multi-levelled GENIAL theoretical framework [6–8,39], focused on individual, collective and planetary wellbeing. (Refer to S2 Text for further details).

#### Mixed methods analysis.

After both datasets were analysed (i.e., quantitative and qualitative), similar concepts from the study’s findings were presented in a side-by-side comparison (Table 3) and examined for convergence and divergence [27].

**Table 3.** Joint display of quantitative and qualitative findings.

Domain	Quantitative Results	Qualitative Findings	Interpretation
Impact of WHM on nature connectedness	Weeks of WHM practice was significantly associated with nature connectedness (*b* =.03, *p* =.033).	**Feeling More Connected:** Practitioners reported an increase in the urge to affiliate with nature, felt a greater sense of interconnectedness with nature, and expressed deeper concern for the natural environment. “It’s just like brought everything to the forefront, I can’t tell you, you know, I’ve walked down [location] and thought ‘oh yeah, it’s just a beach’ but now I feel I just want to be down there.”	Quantitative results revealed a positive relationship between WHM and nature connectedness. Qualitative data reinforce these findings and offer deeper insights into the relationship, emphasising a greater appreciation for nature and concern for its wellbeing since practising WHM.
Impact of WHM on wellbeing	WEMWBS scores were higher in WHM compared to published data on samples recruited prior to (*p* < .001, *d = * 0.78) and during (*p* < .001, *d* = 1.32) the pandemic.	**Cultivation of Positive Psychological States:** Practitioners experienced various positive emotions with the WHM, including calmness, pleasure, and vitality. “I feel exhilarated afterwards. I feel brand new again.” **Feeling More Connected:** Practitioners described feeling a greater sense of connectedness to other people, the natural environment or a higher power. “When I started it, god, we used to argue over stupid things, but we get on so much better now. I feel a big change in how we are with each other, but in a good way.” “It helped me with the fears. Erm and I actually took on volunteer jobs where I was going round to doors giving them bread and stuff like that and, you know, putting a smile on their face, so it helped in many ways, yeah. And it helped me look at people with a bit of love and compassion as well, you know, and understand where they’re at.” “Since I started doing the Wim Hof Method, I’ve managed to become vegan erm which I’ve tried a number of times over the years erm but haven’t been able to do. So, I suppose I have a deeper respect and love for nature. I spend more time in nature and I bring nature into my home...” **Capacity to Cope with Distress and Adversity:** WHM relieved distress and enabled practitioners to cope with various stressors, including the COVID-19 pandemic. “It’s helped me immensely through every single lockdown that we’ve had, believe me. Erm, there’s no way in the world I could have got through them without this […] I will put my hand on my heart and say, probably without those, this might sound really extreme, without this method, I probably wouldn’t be here.”	Quantitative results revealed that WHM has a positive impact on wellbeing. The additional qualitative component provides support for the quantitative results and emphasised the various aspects of wellbeing, spanning individual (i.e., positive emotions and capacity to cope with adversity), collective (i.e., social connectedness) and planetary (i.e., nature connectedness) domains.
Mediating role of nature connectedness	Weeks of WHM practice was not directly associated with wellbeing (*b* =.01, *p* =.240) but was indirectly related to wellbeing via nature connectedness (*b* =.01, *p* < .05).	**Rise of Challenging Climate-Related Emotions:** In one case, feeling more connected to nature generated climate-related distress. “I sort of er started to be more conscious about er not littering on the street, but I’ve never done that, but now when I see that somebody does that I feel it’s very wrong and I just feel pain [...]. I feel pain and I feel a calling to do something […]. I feel that I don’t have enough time […] So I think that’s the reason why I’m going mad because I have a higher, you know, erm like a calling to save the earth and on the other hand I have a real life, “Okay, it needs to wait some years er to do this”, but then I feel that if I can’t act today, then, I don’t know, the world will go crazy. So, sometimes I feel that I’m in the middle and I’m the only one who can help.”	Quantitative results suggest that the longer participants practise the WHM, the greater their wellbeing because they feel more connected to nature. However, qualitative data revealed a more nuanced perspective, showing that stronger connections to nature may also generate climate-related distress. Critically however, WHM helped participants to manage and relieve such distress, indicating its potential in promoting and protecting wellbeing.

## Results

**Fig 2:**
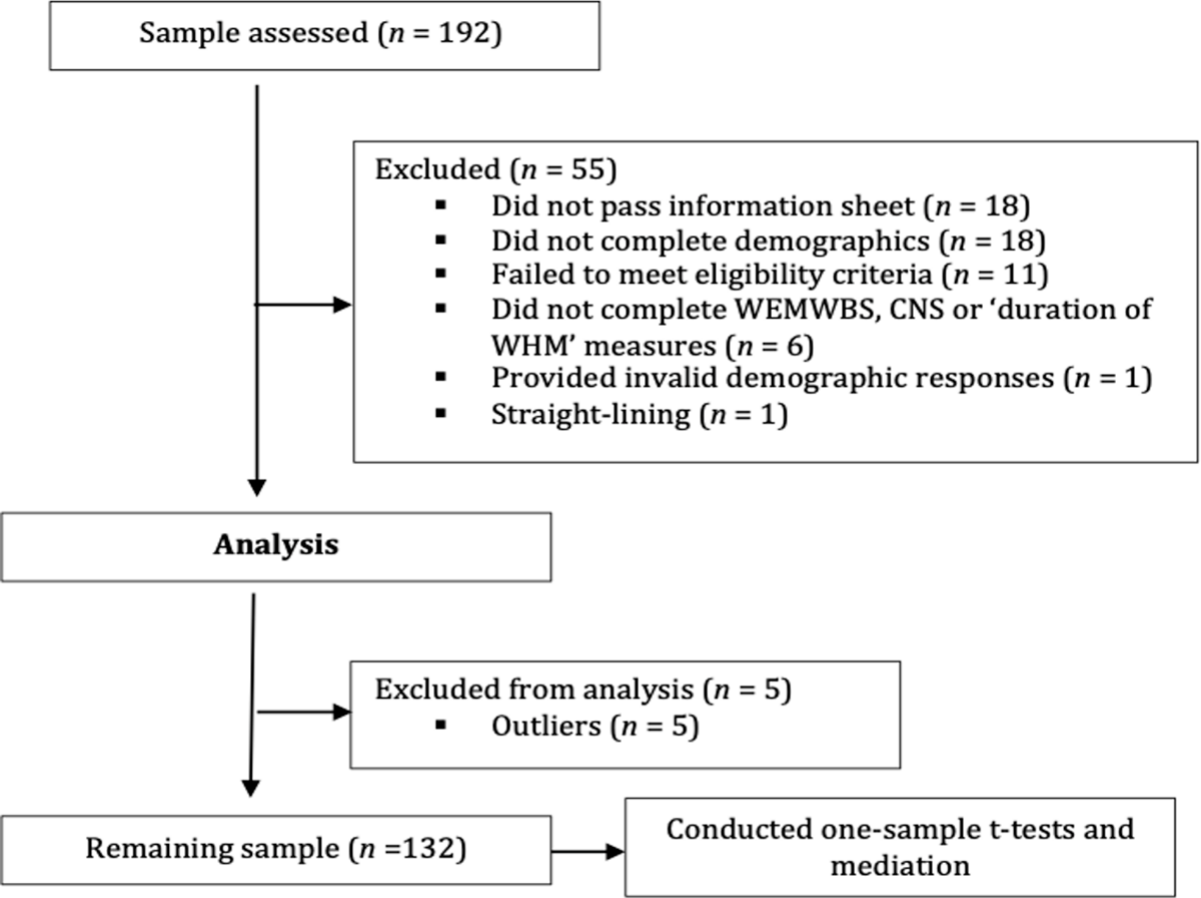
Consort Flow Diagram Depicting the Current Study’s Exclusion Process Prior to Analysis.

A total of 192 participant responses were imported into SPSS, and following exclusions (Fig 2), a total of 132 participants were available for analysis.

### Demographic information

Forty two percent of participants (55 of 132) reported an underlying physical or mental health problem, and 73% (96 of 132) reported that they had been vaccinated with the COVID-19 vaccine. All participants confirmed they were current practitioners of the Wim Hof Method (WHM), 28% (37 of 132) of whom had completed the 10-week fundamental WHM course. (Note that it is still possible to practice the method without having done this course, using for example, the publicly available mobile app and guidance from the many online WHM communities). The majority of participants (96.2%, 127 of 132) practiced the WHM breathing technique and nearly all participants (99%, 131 of 132) engaged in cold-water exposure, with 47.7% (63 of 132) practising both indoors and outdoors. Additionally, 77.3% (102 of 132) practised meditation, 40.9% (54 of 132) engaged in the push-up challenge, and 40.2% (53 of 132) incorporated yoga exercises into their routine.

### Descriptive statistics

Descriptive statistics are presented in Table 1, including Cronbach’s alpha coefficients, medians and interquartile ranges for the CNS, WEMWBS and WHM variables. Median and Spearman’s rank-order correlation coefficients are reported because the CNS and WHM were not normality distributed. Cronbach’s alpha statistics for the CNS and WEMWBS are also presented in Table 2, demonstrating that the scales achieved acceptable to excellent reliability.

**Table 1.** Descriptive statistics, correlations, and cronbach’s alpha statistics for the study variables.

		IQR				
Variable	*Mdn*	Q1	Q3	α	1	2
1. CNS	58.00	53.00	63.00	.71	–	–
2. WEMWBS	55.00	51.00	60.00	.91	.39**	–
3. WHM	35.00	17.50	87.75	–	.17^*^	.08

*Note.* WHM=  weeks of Wim Hof Method practice; CNS=  connectedness to nature scale; WEMWBS=  Warwick-Edinburgh mental wellbeing scale; *Mdn*=  median; IQR*= * interquartile range; Q1=  quartile 1; Q3=  quartile 3; α=  Cronbach’s alpha coefficient.

* *p* < .05 (two-tailed). **p < .01 (two-tailed).

**Table 2.** Main themes alongside number of participants whose comments were associated with each.

Theme	n
Cultivation of Positive Psychological States	14
Capacity to Manage and Cope with Distress and Adversity	13
Feeling More Connected	11
Perceived Vulnerability to COVID-19	5
Moral and Social Responsibility	5
Rise of Challenging Climate-Related Emotions	6
Opportunity for Positive Change	6

### Inferential statistics

#### Wellbeing.

Wellbeing scores were significantly greater in the current sample (*M= * 55.07, *SD= * 7.31) than in the pre-pandemic sample (*M= * 49.4, *SD= * 9.08) [28] (*t*(131)=  8.91, *p < *.001), a finding associated with a medium to large effect size (*d= * 0.78). Wellbeing scores of the current sample were also significantly greater than the sample during-COVID (*M= * 45.4, *SD* not reported) [29] (*t*(131)=  15.20, *p < *.001), a finding associa*t*ed with a large effect size (*d= * 1.32).

#### The role of nature connectedness.

**Fig 3:**
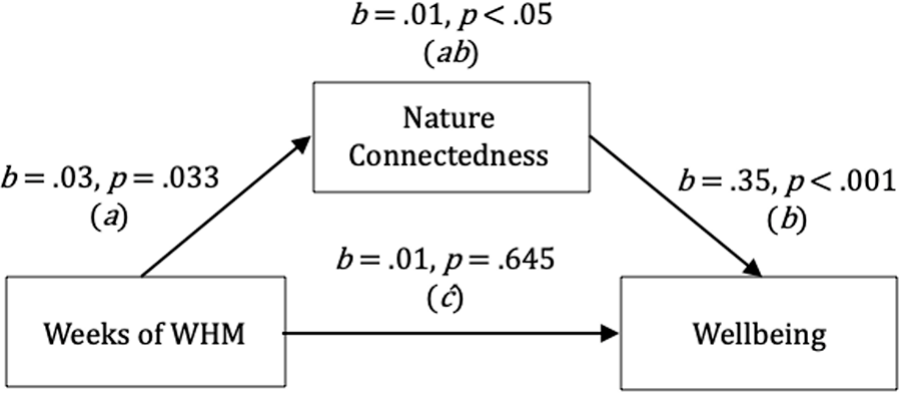
*A Simple Mediation Model Depicting Weeks of WHM Practice Predicting Wellbeing Mediated by Nature Connectedness. Note. ab*=  indirect effect; *c= * the total effect; *c*’=  the direct effect.

The total effect corresponding to weeks of WHM practice predicting wellbeing was not significant, *b* =.01, *p* =.240. The direct effect (weeks of WHM practice predicting wellbeing while adjusting for nature connectedness) was also not significant, *b* =.01, *p* =.645. However, the weeks of WHM practice predicted nature connectedness, a finding that was statistically significant, *b* =.03, *p* =.033, and nature connectedness significantly predicted wellbeing, *b* =.35, *p < *.001. The results also demonstrated a significant indirect (mediating) effect, *b* =.01 [lower limit CI =.001, upper limit CI =.022], indicating that nature connectedness mediated the relationship between weeks of WHM practice and wellbeing (*p < *.05) (Fig 3). Furthermore, in additional analysis these findings were robust to the inclusion of a covariate reflecting the presence of a psychological and/or physical health condition, *b*=.01 [lower limit CI =.001, upper limit CI =.025].

### Qualitative findings

Seven main themes were identified in the thematic analysis (Table 2) and these are described below.

#### Theme one: Cultivation of positive psychological states.

Most participants described the WHM experience as initially challenging and uncomfortable. However, WHM was also reported to evoke feelings of bliss and euphoria, with some describing the experience as drug-like and addicting, suggesting that the WHM cultivates the experience of pleasure:

“It was like really uplifting. You could say it was like a drug, … you felt wonderful, like a really lovely feeling.” (Female, aged 55+, practising WHM for 27-35 weeks, practises WHM indoors and outdoors)

Many participants also reported feeling energised, alert, invigorated and rejuvenated following the practice, suggesting that the WHM promotes feelings of vitality and vigour:

“I think that the exhilaration that I feel and the mental lift that I feel after coming out having just done four rounds of breathing and either a cold shower or some cold exposure in the bath, I feel exhilarated afterwards. I feel brand new again.” (Female, aged 35-44, practising WHM for 1-26 weeks, practises WHM indoors)

The WHM also induced states of calmness and serenity, with participants reporting experiences of indescribable states of deep stillness and peacefulness, experiencing calmness of the mind and being calmer and less troubled by various sources of stress and adversity, including intense occupational demands, climate change and the COVID-19 pandemic:

“I get a calmness and a stillness that is quite alien to me. I feel like it makes me more patient and less reactive, and it kind of seems to settle everything down. Everything becomes a little bit calmer.” (Female, aged 45-54, practising WHM for 53-104 weeks, practises WHM indoors and outdoors)

#### Theme two: Capacity to manage and cope with psychological distress and adversity.

The WHM enabled many participants to manage and relieve psychological distress associated with various stressors, including intense occupational demands, bereavement, childhood traumatic experiences and other major stressful life events, such as the COVID-19 pandemic and the associated lockdown. In one case, it was suggested that the WHM helped one participant cope with and overcome a period of extreme suffering:

“It’s helped me immensely through every single lockdown that we’ve had, believe me. Erm, there’s no way in the world I could have got through them without this with regards to an element of cold or breathwork or mindset […] I will put my hand on my heart and say, probably without those, this might sound really extreme, without this method, I probably wouldn’t be here.” (Male, aged 35-44, practising WHM for 104+ weeks, practises WHM indoors and outdoors)

Many participants reported using the WHM to manage psychological health problems, which enabled them to cope with and relieve periods of low mood and rumination, as well as help reduce symptoms associated with borderline personality disorder, anxiety and depression. Participants often reported having experienced immediate relief from their symptoms, with some participants reporting a full remission of their condition after repeated practice:

“After months I realised that I have no depression. Like, nothing. I felt so good like never in my life.” (Female, aged 35-44, practising WHM for 27-53 weeks, practises WHM indoors)

#### Theme three: feeling more connected.

Many participants described feeling a greater sense of connectedness to other people, the natural environment or a higher power.

Connectedness to others: WHM encouraged interaction with other WHM practitioners through weekend retreats, camping trips, and Christmas day swims, as well as through online groups which provide support and encouragement of WHM practice:

“A Christmas day swim, what an amazing atmosphere. We have one here in [location], and there’d be like about five thousand spectators, possibly two or three hundred people go in the sea, oh, my goodness, it’s just absolutely amazing and everyone’s buzzing.” (Male, aged 45-54, practising WHM for 104+ weeks, practises WHM indoors and outdoors)

Some participants described a noticeable shift in their sense of connectedness to others, with reports of becoming more tolerant and accepting of people with strong opposing beliefs and feeling more connected to co-workers:

“I really enjoyed being in his company, you know, and he still had these ideas, to me that were quite wacky, but, you know, we could talk about it and it was okay because neither of us was sort of trying to beat the other or anything, you know. Er, yeah, I wouldn’t have done that before. That was new.” (Male, aged 55+, practising WHM for 104+ weeks, practises WHM outdoors)

In one case, the WHM was described to have fostered a sense of love and compassion towards others during the pandemic and alleviated their fear of COVID-19, allowing the practitioner to engage in prosocial behaviour:

“It helped me with the fears. Erm and I actually took on volunteer jobs where I was going round to doors giving them bread and stuff like that and, you know, putting a smile on their face, so it helped in many ways, yeah. And it helped me look at people with a bit of love and compassion as well, you know, and understand where they’re at.” (Male, aged 45-54, practising WHM for 104+ weeks, practises WHM indoors and outdoors)

Connectedness to nature: There were reports of an awakened desire to spend time in natural environments, blissful experiences of oneness with nature, having a deeper love and respect for nature, adopting a vegetarian diet, having a heightened awareness and appreciation of natural environments, as well as having an increased tendency to bring nature into the home:

“It’s just like brought everything to the forefront […]. I’ve walked down [location] and thought, “Oh yeah, it’s just a beach” but now I feel I just want to be down there.” (Female, aged 45-54, practising WHM for 27-53 weeks, practising WHM indoors and outdoors)

Spirituality: There were various reports of increased spirituality, including becoming aware of the existence of a higher power, engaging with and reflecting on existential questions about life and reality, transcending the fear of death, recognising a higher calling to save Earth and becoming enlightened about life and past experiences:

“I just think, “Is there anything in it?” I don’t believe, personally, Christianity is correct, I don’t, but there’s something more to us, this much I feel I know now […] I just feel differently. I feel almost like I don’t fear death, perhaps, anymore.” (Male, aged 45-54, practising WHM for 104+ weeks, practises WHM indoors and outdoors)

#### Theme four: Perceived vulnerability to COVID-19.

Many practitioners, both vaccinated and unvaccinated, had a low-risk perception of COVID-19 because they believed their immune system was strong enough to fight the disease. The WHM contributed to this sense of security because of its reported positive impact on the immune system and because practitioners themselves experienced fewer illnesses since practising the method, leaving some to believe that WHM reduces their vulnerability to COVID-19, and therefore, see less need for vaccination.

“It’s [the WHM] helped me feel a little bit more secure and less afraid of erm catching COVID […] I’m not saying that it’s going to stop you catching it. I think probably if you did catch it, you might suffer less or for a shorter period or you might just be generally less likely to pick it up […] So, for me working in [the medical field], I didn’t have a lot of choice, so I had to be vaccinated. Erm, however, I don’t think I’m gonna go and get a booster if I can avoid it because I’m not sure it’s entirely necessary […] I think your immune system works in mysterious ways.” (Female, aged 45-54, practising WHM for 53-104 weeks, practises WHM indoors and outdoors)

In contrast, those who acknowledged the severity of the disease and their susceptibility to it, despite their practice, appeared to be more inclined to accept the vaccine.

“I’m immunocompromised. If I did get COVID, I’m at greater risk of having serious consequences, you know. I know the vaccine would reduce the likelihood of that.” (Male, aged 55+, practising WHM for 104+ weeks, practises WHM outdoors)

#### Theme five: Moral and social responsibility.

Despite a low-risk perception of COVID-19 and a desire to rely on their immune system for protection, many participants chose to have the vaccine primarily to protect other people rather than themselves. This decision was often rooted in a strong sense of social responsibility and a belief in collective wellbeing. Their reasoning extended beyond immediate personal networks to encompass a concern for wider societal impacts, such as reducing pressure on healthcare services and mitigating the broader social consequences of the pandemic:

“I like to think that my immune system, you know, can be strong enough to fight off COVID to the point where it wouldn’t be, you know, really detrimental to my health […] Ideally, I wouldn’t want to have the vaccination, but I feel like I’m doing my bit by doing it […] I’m concerned about women and domestic abuse, well, and men as well. I’m concerned about child abuse. I’m concerned about schools going into lockdown. All these things for me are a greater threat than COVID, but if it’s COVID that’s causing those, then let’s knock COVID on the head […]. That is enough for me to go, “ok I’ll have this vaccination” […] because we need to protect people outside of COVID, vulnerable people.” (Female, aged 35-44, practising WHM for 1-26 weeks, practises WHM indoors)

#### Theme six: Rise of challenging climate-related emotions.

Many participants recognised that humans are causing devastating, unequivocal and irreparable damage to the natural environment, which was a source of significant emotional distress for some, including fear, concern, sadness and pain.

“I just think of all the other animals that are on the planet and even the insects and the mammals. They’re just going about their daily life as they do, and we’re just messing the whole place up, and it is a real worry.” (Male, aged 45-54, practising WHM for 104+ weeks, practises WHM indoors and outdoors)

Some participants were particularly distressed about the consequences that environmental neglect may have for future generations:

“I feel quite terrified. I feel very concerned for the generation behind me, so my children and their children. I feel incredibly sad that we’ve not looked after our planet better than we have.” (Female, aged 45-54, practising WHM for 53-104 weeks, practises WHM indoors and outdoors)

One participant described how feeling more deeply connected to nature cultivated climate-related distress, which was accompanied by a lack of understanding from others, a sense of helplessness, a strong sense of responsibility and environmental concern:

“I sort of, er, started to be more conscious about, er, not littering on the street, but I’ve never done that, but now when I see that somebody does that I feel it’s very wrong and I just feel pain [...]. I feel pain and I feel a calling to do something […]. I feel that I don’t have enough time […] So I think that’s the reason why I’m going mad because I have a higher, you know, erm, like a calling to save the earth and on the other hand, I have a real life, “Okay, it needs to wait some years, er, to do this”, but then I feel that if I can’t act today, then, I don’t know, the world will go crazy. So, sometimes I feel that I’m in the middle and I’m the only one who can help.” (Female, aged 35-44, practising WHM for 27-53 weeks, practises WHM indoors)

#### Theme seven: Opportunity for positive change.

While the lack of climate action was a source of psychological distress for some, there was also a sense of hope and optimism, with some participants reporting that positive changes to the environment are still achievable if appropriate and collective action is taken:

“It worries me that we’re not doing enough, fast enough, and every time I read an article where something is being done, I feel relieved. Er, and then when I hear about other countries not stepping up, or indeed the UK not stepping up enough, it worries me […]. We can turn it around, but it just requires so much.” (Female, aged 35-44, practising WHM for 1-26 weeks, practises WHM indoors)

However, some recognised that efforts to precipitate positive change are needed at multiple levels of scale from the individual to government:

“I’ll try and do the little things where it counts to try and improve the bigger picture, but on a bigger level, it needs to be changed at government level.” (Male, aged 45-54, practising WHM for 27-53 weeks, practises WHM indoors)

Indeed, positive change must be facilitated across multiple scales and domains [40]. Take for example, dietary behaviour. At the individual level, reducing meat consumption and food waste can lower emissions. At the community level, initiatives like local food cooperatives or educational campaigns can promote collective action and shift norms around sustainable eating. At the governmental level, systemic changes such as reforming agricultural subsidies or introducing carbon labelling can reinforce these efforts. While this interplay across scales highlights the potential for meaningful environmental change, coordinating such multi-level initiatives remains a significant challenge due to numerous practical barriers [40].

### Integration of quantitative and qualitative data

Table 3 provides an integration of the quantitative and qualitative data collected in this study.

## Discussion

Confronted with ongoing global threats and stressors, it is imperative to identify interventions that promote wellbeing and resilience at multiple levels of scale. Here, we found that WHM promoted connections to the self, others and the natural world and had a positive impact on wellbeing amid a backdrop of global suffering, which is contextualised in this study by the COVID-19 pandemic. We now reflect on these findings through the lens of the GENIAL model [6–8,39], focused on individual, collective and planetary domains of wellbeing.

### Individual wellbeing

The results indicate that WHM may help to promote a balanced mind [7] evidenced by the cultivation of positive emotions, promotion of meaning and purpose through self-transcendence (e.g., focus on others and nature), and the amelioration of distress. These findings suggest that WHM promotes both hedonic (e.g., pleasure) and eudaimonic (e.g., meaning) aspects of wellbeing [5] while also positively impacting on mental health. This is consistent with a vast body of research demonstrating the psychological benefits of mind-body connection (e.g., breathing techniques) and encouraging contact with nature [16,41].

Consistent with our pre-registered predictions, wellbeing was greater in the current sample than in previous samples [28,29], even exceeding pre-pandemic levels, illustrating the capacity for experiencing wellbeing despite the context of global suffering during the COVID-19 pandemic. This suggests that WHM buffered the negative impacts of the pandemic on wellbeing, potentially owing to its capacity for promoting aspects of psychological resilience, including positive emotions and meaning in life [42,43]. Furthermore, WHM’s influence on COVID-19 risk perception may have also played a role in protecting practitioner wellbeing, given that higher perceptions of risk have been associated with higher psychological distress and lower wellbeing [44]. However, a higher risk perception also predicts more willingness to vaccinate [45], suggesting that by lowering risk perceptions of COVID-19, WHM may have potential psychological benefits but may also inadvertently reduce adherence to disease-preventative interventions such as vaccines. Indeed, the current findings indicate that practitioners with a low-risk perception of COVID-19 were inclined to believe that the COVID-19 vaccine was unnecessary, which has important implications for collective wellbeing if such beliefs translate into vaccine hesitancy.

Overall, these findings provide evidence that WHM can promote and protect individual wellbeing during major adversity. It is important to note that the current results were obtained despite almost half of the current sample (43%) reporting having a psychological or physiological health problem, demonstrating that populations with a relatively high prevalence of distress and impairment also have the capacity to experience wellbeing. Interestingly, the prevalence in our sample is considerably higher than the normative data from England and Wales, where 24% of people reported experiencing impairment (in 2021, the same time data was collected for this study) [46].

### Collective wellbeing

The research found that WHM facilitated connectedness to others and increased the propensity for prosocial behaviour (e.g., volunteering), key determinants of individual wellbeing [47,48]. This may be due to nature exposure, which is known to promote social connectedness and prosocial behaviour [49,50]. Aside from benefiting individual wellbeing, prosocial acts, such as vaccination to protect others, also contribute to collective health and wellbeing [51]. Thus, interventions that facilitate such acts will help to foster safe and resilient communities.

Our findings revealed that WHM practice can enhance the capacity for social connectedness, subsequently increasing concern for the wellbeing of others. However, not all practitioners in the current study were willing to be vaccinated. Some expressed a preference for natural immunity and natural approaches to health through WHM, a stance reflecting a ‘natural-is-better’ bias [52]. This bias reflects the belief that natural interventions, like relying on the immune system, are inherently safer or healthier than medical alternatives such as vaccines. Critically, this bias has been shown to influence vaccine-related decisions [53] and may be heightened in those who are more connected to nature [26]. Consequently, WHM’s role in cultivating nature connectedness may have inadvertently reinforced this bias, which in turn may have contributed to vaccine hesitancy. This may help to explain why vaccine acceptance (including those who intended to be vaccinated), although relatively high, was still lower in the current sample (73%) than in the wider UK population (96%) [54].

Taken together, these findings suggest that WHM can positively influence collective wellbeing by encouraging prosocial behaviour but may also discourage behaviours (e.g., vaccination) that are critical for collective health and wellbeing in some individuals. However, it is important to note that some practitioners were vaccinated to protect others despite believing that natural immunity is superior, suggesting a willingness to prioritise collective wellbeing over individual preferences and self-interests. This tension between a desire for natural immunity and prosociality may have fostered a difficult yet critical decision-making process among the participants. While some may have navigated this tension by strictly adhering to their ‘natural’ approaches to health, others appeared to have transcended such preferences, opting for vaccination in the interest of public health and wellbeing. Interestingly, acting in selfless ways have been described as one of the deepest levels of meaning [55] and have been linked to increased positive affect [56] and lower distress [57], and may, therefore, have had a positive impact on the practitioners’ wellbeing.

### Planetary wellbeing

The findings suggest that interacting with nature during WHM practice contributed to wellbeing. Indeed, practising WHM in natural environments evoked positive, often indescribable psychological states, consistent with Maslow’s [58] ‘peak experience’. In one instance, WHM led to the development and pursuit of a higher purpose to save the planet, indicating a commitment to planetary wellbeing and providing compelling qualitative support for the quantitative impacts of WHM on connection to nature. This aligns with previous findings showing that engaging in nature-based activities and feeling connected to nature significantly predict the promotion of hedonic and eudaimonic markers of wellbeing, such as happiness and purpose in life [20,59]. Furthermore, such intense psychological experiences in nature have also been shown to support planetary wellbeing by encouraging engagement in pro-environmental behaviours [60], similar to reports from those interviewed. These findings highlight the multifaceted benefits of WHM and its potential for promoting both individual wellbeing and environmental health.

Both the quantitative and qualitative strands of the study revealed that WHM enhances a sense of connectedness to nature. The quantitative results showed that this enhanced connection mediates the relationship between WHM and wellbeing, suggesting that the longer participants practise WHM, the greater their wellbeing and that this increased wellbeing is underpinned by their sense of connection to nature. However, the qualitative insights introduced a layer of nuance, revealing that WHM can also generate climate-related distress. The qualitative findings also revealed that WHM improved the capacity to manage this distress, indicating that the method not only acts as a catalyst for heightened nature connectedness and environmental awareness but may also equip practitioners with the resilience necessary to cope with the psychological challenges that such connectedness and awareness can bring [61].

Distress and suffering can be a catalyst for wellbeing and are not necessarily an impediment to it. Critically, negative emotional responses to climate change are known catalysts for climate action [61], and participants in the current study were hopeful about combating the climate crisis despite it being a source of distress. This sense of hope has also been associated with intentions to engage in climate action [62]. Thus, interventions that cultivate and support the development of active and constructive hope may provide a promising pathway to planetary wellbeing. These findings therefore have important implications for the intersection of population and planetary wellbeing.

### Strengths and limitations

The greatest strength of the present study was the mixed methods approach, providing both breadth and depth of understanding relating to wellbeing in the WHM population. The quantitative data allowed for the testing of predictions in a sizeable sample, considering the uniqueness of the population and the specific period in which the study was conducted – during COVID-19. It also enabled the robustness of findings to be determined through bootstrapping and additional analysis. Meanwhile, the qualitative data enriched our understanding of the potential mechanisms behind the observed associations and offered more nuanced insights into the impact of WHM on wellbeing. While the cross-sectional nature of this study limits our ability to infer causality, it has laid the foundation for future investigations that could offer stronger evidence for causal relationships (e.g., randomised controlled trials).

Regarding the mediation model, correlational analysis indicated that the relationship between the predictor (X, WHM) and the outcome (Y, WEMWBS) was not significant. Traditionally, this would imply that mediation cannot be tested, however, a significant direct effect between the predictor (X) and outcome (Y) is no longer a prerequisite for mediation analysis [63, 64]. Instead, mediation can be tested as long as there is a significant relationship between the predictor (X) and the mediator (M), and between the mediator (M) and the outcome (Y). In this study, WHM (X) was significantly correlated with the connectedness to nature scale (CNS) (M), and CNS (M) was significantly correlated with WEMWBS (Y), which supports testing the indirect effect of WHM on wellbeing through CNS.

Another strength pertains to the unique timing and context of our study. Conducting the study during a period of global hardship and suffering amplifies the relevance and applicability of our findings to current and future challenges. However, the timing of our study coincided with the easing of the COVID-19 lockdown in the UK [65], so it could be argued that our findings of heightened wellbeing could reflect post-lockdown relief rather than the impact of WHM. Nonetheless, the observed levels of wellbeing surpassed pre-pandemic benchmarks, and qualitative insights revealed that participants attributed their enhanced wellbeing directly to their WHM practice. Together, this suggests that the improvements in wellbeing are indeed a result of WHM, and the lift in lockdown restrictions is unlikely to have underpinned these findings.

Finally, the timing and context of our study also meant that our final sample size was slightly lower than originally intended. We calculated an a priori sample size calculation and aimed for 156 participants; however, the context and timing of the study—focused on Wim Hof Method practitioners during the COVID-19 pandemic—led to a final sample of 132 participants. We believe our findings remain robust for several reasons. First, our analysis was pre-registered, ensuring transparency and reducing the risk of bias in our approach. Second, we used bias-corrected bootstrapping, a method known for providing accurate confidence intervals in mediation analyses, even with smaller samples. Third, the observed indirect effect was statistically significant, reinforcing the robustness of the mediation pathway reported here. Fourth, the findings align with existing theoretical models and literature on nature connectedness and wellbeing, adding confidence to their interpretation. Finally, the integration of both quantitative and qualitative data reinforces the robustness of our findings.

In summary, these considerations suggest that, while not without limitations, the current study offers meaningful contributions to understanding the role of WHM in enhancing wellbeing, particularly during major societal challenges such as COVID-19.

### Directions for future research

While it is evident that WHM positively impacts wellbeing, there is much variety in how practitioners carry out the practice and begs the question as to whether particular components of the method are responsible for the observed effects. Regardless, our findings emphasise that learning to embrace the physical and psychological discomfort generated by WHM may be key to developing psychological toughness and the experience of wellbeing in the face of significant hardship and suffering. This is an area that would benefit from further investigation.

## Conclusion

In summary, this study is the first to show that WHM can enhance wellbeing while fostering a sense of connection to self, others and nature. By integrating cold exposure, breathwork, meditation, and exercise, WHM deepens engagement with the environment. Notably, WHM practice significantly predicted nature connectedness, which in turn predicted wellbeing. This strengthened bond with nature may heighten environmental awareness, positioning WHM as a potential tool for addressing climate challenges through the lens of wellbeing.

## Supporting Information

S1 Text Interview topic guide. (DOCX)

S2 Text Additional methodological detail for quantitative and qualitative analysis. (DOCX)
