# Supplementary material for: Wellbeing, nature connection and vaccine attitudes: A convergent mixed methods study in Wim Hof Method practitioners
Source: PLOS Ment Health. 2025 Mar 26;2(3):e0000281. doi: 10.1371/journal.pmen.0000281 (PMC12798517; doi:10.1371/journal.pmen.0000281)
Supplement: S1 Text — (DOCX) [file pmen.0000281.s001.docx]

**S1 Text**

**Interview Guide**

1. **WHM Practice:**

- What were your reasons for starting the WHM?
- How would you describe your experience with the WHM?
- What do you enjoy the most about the WHM?
- How would you describe your health and wellbeing since practising the WHM?

1. **COVID-19 Pandemic and Vaccine Choice:**

- Would you say that the WHM has helped you during the pandemic? If so, how?
- What has it been like for you living in a pandemic with the threat of COVID-19?
- What do you feel is the best way to protect yourself from COVID-19?
- What are your reasons for choosing to have, or not have, the COVID-19 vaccine?

1. **Nature Connection:**

- How connected with nature do you feel?
- Has your connection with nature changed since you started practising the WHM?
- There is some indication that people who are more connected to nature are more likely to engage in pro-environmental behaviours. What do you think about that?
- How do you feel about the climate crisis?
