# Supplementary material for: Wellbeing, nature connection and vaccine attitudes: A convergent mixed methods study in Wim Hof Method practitioners
Source: PLOS Ment Health. 2025 Mar 26;2(3):e0000281. doi: 10.1371/journal.pmen.0000281 (PMC12798517; doi:10.1371/journal.pmen.0000281)
Supplement: S2 Text — (DOCX) [file pmen.0000281.s002.docx]

**S2 Text**

**Analysis**

***Quantitative***

All survey responses were recorded on Qualtrics and exported into SPSS (Version 28) for analysis. Data entries that were incomplete (i.e., failed to answer all questions), included invalid responses (e.g., answered ‘99’ to all demographic questions), or evidenced straight-lining were removed prior to analysis. Z scores were calculated on WEMWBS, CNS, and ‘weeks of WHM’ variables to identify outliers (i.e., data entries that fell beyond three standard deviations from the mean), which were then removed to prevent obtaining misleading results (Tabachnick & Fidell, 2014). Two one-sample t-tests were then performed using SPSS, and after ensuring that the assumptions for mediation analysis were met (Hayes, 2022), a simple mediation analysis (model 4) was performed using PROCESS (Version 3.5) for SPSS.

***Qualitative***

The follow-up interviews were audio recorded and analysed using reflexive thematic analysis following the guidelines suggested by Braun and Clarke (2006, 2012, 2021). Thematic analysis was used because it is flexible in its application regarding underlying philosophical frameworks (Terry et al., 2017), aligning well with the pragmatic paradigm guiding this study. Since the WHM is a relatively unexplored population, particularly in terms of wellbeing, data were coded inductively, after which findings were interpreted within the context of the multi-levelled GENIAL theoretical framework, focused on individual, collective and planetary wellbeing (Isham et al., 2023; Kemp et al., 2017; Kemp & Fisher, 2022; Mead et al., 2021).

To begin the analysis, all audio recordings were transcribed verbatim and were double-checked for accuracy. Next, transcripts were thoroughly reviewed and explored multiple times for familiarisation and were then imported to NVivo 12 software for data management. The interview data was subjected to three rounds of coding (i.e., starting with interview one, then working through the dataset to interview fifteen, then working backwards through the dataset, then working through the dataset in random order). The coding process was carried out by one researcher, consistent with the latest guidelines for reflexive thematic analysis (Braun & Clarke, 2021).

After coding, the focus shifted to theme generation and development, which involved organising and clustering (and re-clustering) groups of codes around a shared idea or concept. Next, the transcripts were re-read to identify potential codes that were missed during the previous stages of analysis, and the themes were compared against the entire dataset to determine if they reflected the data content to prevent misrepresenting the findings. Lastly, all themes were defined and assigned a name that captured the essence of the data situated within each theme. Please note that the data extracts that are included in this report have been edited to enhance their readability. Unnecessary detail has been removed and replaced with an ellipsis. Repeated words or phrases have also been removed (Braun & Clarke, 2021).
